# Use of whole genome sequencing for surveillance and control of foodborne diseases: *status quo* and *quo vadis*

**DOI:** 10.3389/fmicb.2024.1460335

**Published:** 2024-09-13

**Authors:** Tristan Schadron, Maaike van den Beld, Lapo Mughini-Gras, Eelco Franz

**Affiliations:** ^1^Centre for Infectious Disease Control, National Institute for Public Health and the Environment (RIVM), Bilthoven, Netherlands; ^2^Institute for Risk Assessment Sciences, Utrecht University, Utrecht, Netherlands

**Keywords:** surveillance, foodborne disease, source attribution, outbreak detection, whole-genome-sequencing

## Abstract

Improvements in sequencing quality, availability, speed and costs results in an increased presence of genomics in infectious disease applications. Nevertheless, there are still hurdles in regard to the optimal use of WGS for public health purposes. Here, we discuss the current state (“*status quo*”) and future directions (“*quo vadis*”) based on literature regarding the use of genomics in surveillance, hazard characterization and source attribution of foodborne pathogens. The future directions include the application of new techniques, such as machine learning and network approaches that may overcome the current shortcomings. These include the use of fixed genomic distances in cluster delineation, disentangling similarity or lack thereof in source attribution, and difficulties ascertaining function in hazard characterization. Although, the aforementioned methods can relatively easily be applied technically, an overarching challenge is the inference and biological/epidemiological interpretation of these large amounts of high-resolution data. Understanding the context in terms of bacterial isolate and host diversity allows to assess the level of representativeness in regard to sources and isolates in the dataset, which in turn defines the level of certainty associated with defining clusters, sources and risks. This also marks the importance of metadata (clinical, epidemiological, and biological) when using genomics for public health purposes.

## 1 Introduction

Public health infectious disease surveillance entails the systematic collection, analysis, and interpretation of data related to the occurrence and spread of infectious diseases. In this way, trends can be analyzed and outbreaks can be detected. An outbreak is defined as an unusually large number of patients with a specific disease, pathogen or strain linked to each other and/or to a common source of infection. The detection of these outbreaks includes the subsequent identification and characterization (for example antimicrobial resistance or virulence patterns) of pathogens. In the case of zoonoses, and foodborne zoonoses in particular, genomic surveillance and monitoring data from the human and animal domains provide the opportunity to closely monitor the characteristics of circulating strains and infer risks, sources, and transmission routes. In this regard, genomic surveillance is a pivotal means of protecting public health, by taking proactive measures to identify hazards.

Historically, a variety of phenotyping and genotyping approaches have been applied to perform surveillance of foodborne infections (for a review on the detection of different foodborne pathogens, see Aladhadh, 2023). However, in recent years, whole-genome sequencing (WGS) has turned into a standard for surveillance where available resources allow it. WGS has been shown to be a “one-size fits all” superior typing method for surveillance but also for closely related tasks, such as source attribution and hazard characterization (a discipline rooted in microbial risk assessment) (den Bakker et al., 2014; Dallman et al., 2015). This is due to the increased resolution as compared to previously used molecular typing techniques. This is not to say that WGS does not have limitations.

In order to characterize the data, however, the isolates need to go through a genomics pipeline, which includes a library preparation, sequencing and post-sequence processing.

Many of the sequenced genomes consist of short-reads. Consequently, there are gaps where low sequence diversity, regions filled with repeats and low coverage is present. Within these uncaptured sequences a variety of genes may be present. For example, incorporated phage DNA is often flanked by low sequence diversity regions and consequently difficult to map. In addition, sorting out plasmids may be difficult due to repetitive regions and inversions, while important genes may be present on plasmids. For example, the spv operon may be plasmid-encoded on *Salmonella* increasing invasiveness and host cytotoxicity (Guiney and Fierer, 2011). Long-read sequencing offers an alternative able to overcome these problems, but has not been widely implemented yet in current surveillance systems.

**Figure 1:**
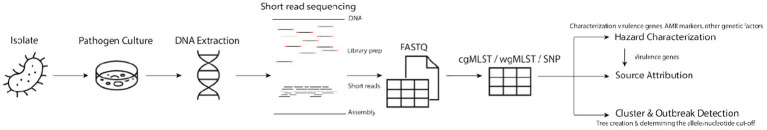
Workflow of the analysis to detect and characterize foodborne pathogens.

The widespread application of WGS data for foodborne pathogen surveillance in general only became possible due to a decrease in sequencing costs and time, increased computational capacity and radical improvements in the accuracy of WGS (Franz et al., 2016). WGS is able to discover links between seemingly isolated incidents where less discerning methodologies cannot. Therefore, many surveillance systems currently implemented WGS as the norm (Brown et al., 2019). Nevertheless, there are still hurdles in regards to the optimal use of WGS analysis for public health purposes. Here, we discuss the current state (“*status quo*”) and future directions (“*quo vadis*”) of these new techniques to improve cluster/outbreak detection, hazard characterization and source attribution of foodborne pathogens (Figure 1; Table 1).

**Table 1:** Comparison of the virtues and pitfalls of using whole genome sequencing methods.

Virtues	Pitfalls
Increased resolution/accuracy compared to molecular typing techniques.	Requires tags to sequence, which requires knowing about the isolate prior to sequencing.
Metagenomics allowed culture-independent sequencing.	Short read sequencing may not be able to resolve low sequence diversity regions.
Long read sequencing may be able to partially resolve low sequence diversity regions.	Long read sequencing until recently not accurate enough, as such not widely implemented yet.
Lack standardization does not seem to significantly impact resulting outbreak clusters.	Lack standardization regarding sequencing platforms and post sequencing data handling pipelines.
Allows production quantitative amounts of data on which a variety of analyses can be performed.	Quantitative data may form a bottleneck in further analyses.
Metagenomics can capture community level compositions.	Metagenomics approaches disregard individual pathogens.
Easier comparability between labs, reanalysis and storage compared to culture-based antibiotic tests.	Individual pathogens must be present in high enough numbers to be able to be detected through metagenomics.
Larger amounts of quantitative data allow for stronger inferences about associations with GWAS.	

## 2 Clustering and outbreak detection

A variety of techniques is used for public health surveillance of foodborne diseases. Most widely applied is case-based passive surveillance, meaning that a certain fraction (i.e., the more severe) of symptomatic cases are microbiologically diagnosed and centrally reported. Frequently, the isolated pathogen is further typed and characterized. Here, a strong shift has been made from phenotypic (for example serotyping using antisera) and low-resolution molecular methods [for example, 7-locus multi-locus sequence typing (MLST), Pulsed-field Gel Electrophoresis (PFGE), and multiple locus variable-number tandem repeat analysis (MLVA) typing] (Maiden et al., 1998; van Belkum et al., 1997; Swaminathan et al., 2001) toward whole-genome-sequencing (Atxaerandio-Landa et al., 2022). Traditionally, outbreak detection was based on the observed number of cases with a certain pathogen (or subtype/serotype based on the lower-resolution typing methods) in relation to the expected number based on time-series analysis of the surveillance history. With the high-resolution power of WGS, this has shifted toward the detection of clusters of cases with closely genetically related strains (defined by a threshold in genomic difference). In genomic surveillance of foodborne diseases, the assumption is that such clusters of high similar strains share a common source that it aims to investigate and eliminate. These clusters can occur without exceeding the total number of (expected) cases of specific pathogen or subtype/serotype in a specific timeframe.

When focusing purely on genetic relatedness, other genomic features defining a strain are ignored. To illustrate, within a healthcare setting plasmids and antimicrobial resistance genes are routinely monitored and evaluated to trace specific strains. In a paper by Joseph et al. (2020), during a *Campylobacter* outbreak strains were defined by analysis of both alleles and antimicrobial resistance genes. However, not in all settings is additional experimental information available.

The commonly applied methods for detecting these clusters based on WGS are core-genome multi-locus sequence-typing (cgMLST) and single nucleotide polymorphisms (SNP) typing. However, there exist other techniques based on genomics data, such as whole-genome MLST (wgMLST) and kmer approaches. The cgMLST approach utilizes a large set of core genes, common to every isolate of the samples, where sequence diversity within this set of genes (“alleles”) provides the basis for comparison of strains (Mellmann et al., 2011). wgMLST includes, besides the core loci, a large repertoire of accessory loci, which are not common to all isolates (Sheppard et al., 2012). In contrast to these allelic approaches, SNP typing uses individual nucleotide differences to discern isolates from one another. A core of positions can be considered as well, which is covered by all query genomes, called the core SNP (Uelze et al., 2020). Lastly, K-mer based approaches divide genomic data into parts of equal length called K-mers (Compeau et al., 2011). As such, K-mers are not necessarily limited by reliance on references, such as the abovementioned typing approaches excluding SNP (Harris, 2018).

All these approaches have become possible with the advent of WGS, but differ primarily in resolution. There is no consensus on preferred use, and this may differ per country or institute. For example, the Netherlands and Denmark commonly apply allelic approaches, such as cgMLST, while France and the United Kingdom favor a nucleotide approach (SNP) (Dallman et al., 2015; Schjørring et al., 2017; Coipan et al., 2022). However, allele-based approaches are the preferred analysis for foodborne pathogens in Europe. To illustrate, for *Salmonella*, allele-based approaches were used by 82% of the member states in a study, whereas only 18% applied a nucleotide-based approach (Van Den Beld et al., 2023). In addition, there is a considerable diversity and lack of standardization regarding sequencing platforms and post sequencing data handling pipelines. Altogether, it may be expected that these differences hamper a uniform assessment of surveillance and outbreak data (for example placing cases in or outside clusters). However, it has been demonstrated that the resulting clustering is surprisingly robust (Pearce et al., 2018; Coipan et al., 2020; Szarvas et al., 2021). This is relevant for situations, which require cross-border cooperation such as international outbreaks.

An illustration of the benefit of harmonized multi-nation corporation, is the large-scale *Salmonella enterica* serovar Enteritidis outbreak related to the consumption of eggs originating from Poland, which could only be related back to its’ origin of contamination through outbreaks in multiple countries and the subsequent transfer of important information including harmonized WGS data and analysis (Pijnacker et al., 2019).

Surveillance and outbreak detection is performed through cluster analysis of WGS data, which is based on genomic distances between isolates. Phylogenetic methods show the genetic relationship between isolates, where bootstrap values in branches indicate the statistical support for the accuracy of that branch. Generally, clustering is performed either on allele-level or nucleotide-level, for which a distance matrix or profile is constructed.

In practice, institutes that apply nucleotide/allele-based clustering apply some form of genomic-distance cut-off to identify clusters, where clusters are made up of isolates with fewer nucleotide/allele differences between them than the cut-off.

### 2.1 Overcoming the nucleotide/allele-threshold

There are problems with setting a fixed threshold, since doing so disregards the nature of the pathogen and outbreak in question. High heterogeneity among characteristics in both pathogens and the epidemiology alters the cluster composition (Duval et al., 2023). Pathogen characteristics hampering correct cluster detection include horizontal gene transfer (HGT) (Arnold et al., 2022) and high levels of genome plasticity (Woodcock et al., 2017), but also the existence of thoroughly conserved genomes. Thus, the context of the pathogen population structure is an important aspect in defining thresholds. Currently, historically proven epidemiological related cases are used to optimize thresholds. In reality, pathogen behavior can differ greatly between strains/serovars/subspecies through space and time, potentially complicating the retrospective use of confirmed outbreaks to set a cut-off threshold. For example, mutation, substitution, and recombination rates may vary vastly due to differences in ecology and population biology (Barrick and Lenski, 2013). These characteristics can also be dynamic in place and time due to different and/or changing selection pressures that are encountered by the same pathogen species (or serovar/serotype) in different niches. Combinations where both the number of SNPs/alleles and the bootstrapping values determine SNP/alleles cut-offs within groups (Pightling et al., 2018) or variable SNP/allele thresholds through time (Payne et al., 2021) mitigate part of the problem. However, they do not fully account for the evolutionary dynamics underpinning the threshold, such as horizontal gene-transfer and within-host/farm evolution. All in all, a non-variable cut-off rate may not be the best approach to surveillance.

Several modeling approaches have been suggested and/or applied to overcome the problems associated with fixed genomic distance threshold for cluster definition. Other approaches predict the SNP/allele cut-off for a particular pathogen (Coll et al., 2020; Dallman et al., 2021).

In recent years, a number of modeling studies have incorporated the mutational rate and time to more realistically capture changes in the number of SNP differences between those isolates within the outbreak clusters and outside of the cluster. For example, Octavia et al. (2015) incorporated the mutational rate of a *Salmonella enterica* serovar Typhimurium type, using differences in MLVA (Octavia et al., 2015). The study included short-lived point-source outbreaks, but this may not always reflect reality. Inclusion of a parameter of time would further allow specifying the amount of acquired mutations.

In a modeling study by Duval et al. (2023), they realize the incorporation of time by defining the duration of the outbreak in addition to specific mutation rates. In this way, the study simulates bacterial evolution to estimate the various genetic distance thresholds of strains for point-source single-strain food or environmental outbreaks (Duval et al., 2023). In practice, the initial onset of the outbreak is often uncertain. The benefit of a model is that the time since source contamination can be estimated, besides the estimation of the mutational rate, thus filling in these knowledge gaps.

In essence, Duval et al. (2023) and Octavia et al. (2015) overcome problems related to the fixed threshold by creating dynamic thresholds, but require the use of data to create a model for this purpose. Another approach was taken by Payne et al., whom applied two different cluster definitions, one including a stringent SNP cut-off, while the other included all samples falling within the maximum number of SNPs differences as observed within a 4-week period. This is aimed at keeping sensitivity and specificity high, without sacrificing one for the other. However, in reality the length of an outbreak can differ considerably, some persisting for long periods of time. Therefore, a rigid time cut-off may not be the best approach.

All in all, though the previously mentioned approaches provide means to overcome problems associated with clustering, the extra data or metadata necessary for these methods to work may not be present. In such cases, the threshold approach provides the best pragmatic way.

### 2.2 Machine learning approaches for surveillance

Machine learning (ML) is able to “learn” to recognize patterns within large datasets, using the information within the patterns to predict other sets of data. There is a large number of different machine learning approaches, such as random forest (Tin Kam, 1995), neural networks, support vector machines, and clustering techniques. These can broadly be subdivided into unsupervised and supervised machine learning methods. Unsupervised models cluster together similar isolates, without knowledge about their class. Whereas, supervised models “learn” on a training set by classifying the input features (allelic/nucleotides) based on a predetermined class (host source). After the training phase, a test set can be used to determine the ability of the model to correctly predict a class for a sample. From this, the probabilities of each isolate belonging to a particular class of pathogens (e.g., cluster or clade etc.) is retrieved (this concerns categorical ML, not regression). Supervised ML thus differs in how models are constructed and how classification is performed. The idea behind using ML methods for clustering is that they help uncover patterns, which are not readily apparent to the naked eye, of genes/mutations associated with a particular class. From the constructed model, feature importance can be retrieved. Furthermore, the time required to perform ML is considerably less than uncovering these patterns manually. Notwithstanding the benefits of ML, during this process many things need to be taken into account. Among others, overfitting, dataset imbalances, the dimensional space (for this feature reduction may be applied, through dimension reduction, dissimilarity analysis and Boruta functions) (Munck et al., 2020), but also method-related hurdles, such as scalability to increasing amounts of data.

Whole-genome sequencing data can be used as input data, either through SNP or kmer frequencies, cgMLST or wgMLST. The application of unsupervised ML for cluster delineation has been evaluated for *Salmonella Enteritidis* in a paper by Coipan et al. (2020). They found that clusters of isolates concordant to one another could be found using unsupervised ML (Coipan et al., 2020). Nevertheless, clusters are determined through stringency, which in the end is based on a predefined number of clusters. This requires prior knowledge about the population structure, transmissions and the number of outbreak clusters.

### 2.3 Network analyses

Network approaches allow for another alternative (Sanaa et al., 2019; Cori et al., 2018) for identifying clusters. The genetic link between isolates could be represented through the genetic relatedness being shown as a weighted link. Similar genomes have smaller distance values, so cluster coherence of outbreak clusters together with the driving forces of parameters in the formation of clusters, can be evaluated. GenomeGraphR, a web-application for foodborne pathogen WGS data analysis is able to construct a graph linking isolates together (Sanaa et al., 2019). Nevertheless, the connectivity is based on a SNP threshold. Additionally, it is assumed that the entirety of the population is sampled, although this is rarely the case. As such, bias is introduced, which some network approaches attempt to circumvent through the introduction of an unknown fraction (Merlotti et al., 2020).

## 3 Source attribution

Source attribution consists of partitioning human cases caused by foodborne pathogens to their putative source of infection. The sources these cases are subdivided into denote not only reservoirs, such as animals, but may also include transmission pathways (e.g., food, the environment, contact with animals, etc.), exposure (e.g., meat, eggs, water, etc.), and risk (e.g., consumption of raw meat, swimming in surface water, petting a dog, etc.) (Mughini-Gras et al., 2019). Genotypic information of isolates retrieved from humans and (animal/food/environmental) sources is compared in order to infer their (most likely) origins. The overlap and differences in data are therefore critical in discovering the relationship between isolates retrieved from humans to a potential source. Since similarities or the lack thereof in sequence data carry informational data, which may hint to relatedness between two isolate genomes.

Genotypic separation of isolates or lack thereof, however, often provides difficulties for source attribution. This is due to the pathogen behaviors and evolutionary dynamic forces shaping genomic changes, and mutability of the genomes themselves. For example, a specialist lifestyle conforms to evolutionary pressures to adapt specifically to that single host, whereas a generalist may benefit from higher amounts of mutability to quickly adapt to changing hosts. In general, host switching behavior and its frequency can erode specific genomic signals, which can make surveillance and source attribution more difficult (Dearlove et al., 2016; Woodcock et al., 2017). Other pathogen characteristics may also play a role, such as genome plasticity (Woodcock et al., 2017), host range (Sheppard et al., 2014), pathogen population sizes and structure (Pightling et al., 2018), and horizontal gene transfer (Arnold et al., 2022). Various other aspects, such as the ability of a host to contribute to human infection, and lack of inclusion of spatio-temporal patterns, can contribute to the erosion of genomic signals as well (Smid et al., 2013). Therefore, disentangling the overabundance or absence of differences within the genomic isolates, to extract information relating to the attribution of these isolates to a specific host is the primary difficulty currently faced while performing source attribution.

In order to improve separation of pathogen isolates originating from different sources, a more complete capture of genome differences is ideal. However, current sequencing is primarily done with short-read sequencing, which is unable to capture low sequence diversity, regions containing repeats, and low coverage areas. The continuous improvement of long-read sequencing will undoubtedly in the future allow for more correct nucleotide calling, with an acceptable number of errors (Amarasinghe et al., 2020). The additional information may help elucidate patterns of information, e.g., through differences in number of repeats and through capture of cases of horizontal gene transfer, which are flanked by regions of low sequence diversity.

### 3.1 Machine learning approaches for source attribution

In recent years, the application of ML for source attribution has been widespread, making use of many techniques, and even comparing different methodologies (Arning et al., 2021; Lupolova et al., 2019; Munck et al., 2020; Brinch et al., 2023; Tanui et al., 2022). Overall, ML algorithms are able to discern differences within the data, where classical typing approaches have not. Nevertheless, where strains are ubiquitous across sources, ML techniques struggle.

Unsupervised ML methods consist of various clustering and dimension reduction techniques. Both clustering and dimension reduction try to group the data. During dimension reduction, the number of features are thereby compressed, while this is not the case in clustering (Lupolova et al., 2019). Generally, the performance of unsupervised ML methods is negligible compared to supervised ML approaches. This is due to supervised ML being guided by class toward relevant patterns of information, whereas unsupervised ML is not. However, this requires predefined classes. Therefore, the existence of an undetermined class could cause a faulty model.

Among the supervised ML methods, one of the more commonly applied methods for source attribution is random forest (Fu et al., 2022; Zhang et al., 2019). Random forest (RF) consists of an assembly of decision trees. Where every tree makes a decision determining the source of the isolate. RF requires little tuning. In addition, the ease of extraction of relevant features and comparatively good performance has propelled its popularity.

However, the ensemble of decision trees can improve only through chance on previous iteration, and categorizes samples based on majority voting. Therefore, a gradient boosting assembly method may be the preferential choice, as the local topography of any given tree is explored, to see if a better performance can be achieved (Mason et al., 1999).

Moreover, the underlying nature of the input features needs to be considered. To illustrate the underlying nature, host range is one of the issues plaguing correct attribution. A generalist lifestyle generally means that source-specific signals associated with a particular host are mostly absent. Without high resolution, this makes attribution more difficult. Meaning that segregation might be considerably less, complicating source predictions (Arning et al., 2021). This is especially the case when the dimensional space is less.

Most techniques primarily consider the cgMLST (Tanui et al., 2022; Munck et al., 2020); however, kmer frequencies are also applied (Arning et al., 2021; Brinch et al., 2023). Studies comparing ML approaches, consistently find gradient boosting approaches to be the best predictors (Arning et al., 2021). This seems to be coupled to the use of cgMLST, where there is a lower resolution and consequently a lower dimensional space. Therefore, the explorative nature of these studies seems only relevant when considering the cgMLST genotyping approach and cannot be extrapolated to higher dimensional datasets. In case of wgMLST, where a larger feature space is involved, there have only been limited attempts made to perform machine learning (Gu et al., 2023). Meaning that studies exploring the performance in combination with wgMLST or the entirety of the genome, where a larger feature space is involved, are required. Nevertheless, in cases of host-restricted pathogens with very conserved genomes, and where a good cgMLST scheme is known, cgMLST approaches may be sufficient.

Feature space and sample size need to be considered, e.g., support vector machine (SVM) perform well in low and high dimensional spaces, but become overshadowed by other techniques when moving toward a higher amount of data (Ghaddar and Naoum-Sawaya, 2017). This is primarily due to memory intensity of the algorithm. Deep learners, using neural networks (NNs), on the other hand, generally improve when the complexity within the data becomes bigger (Hopfield, 1982). The move toward long-read sequencing and higher resolution genotyping or nucleotide schemes, with the increases in dataset sizes, may thus facilitate a move toward the use of these algorithms.

Nevertheless, NNs are seen as somewhat of a “blackbox.” This means that relevant features within the data are not readily apparent. While the extraction of these features is more difficult, it may be required when the feature space needs to be shrunk. For example, the feature space is shrunk in order to boost predictions with the use of more specific relevant features. Feature importance is essential for this, but other techniques, such as removing redundant features/identical strains and dimension reduction may also be applied. Besides, feature reduction may be done by another ML approach before inputting a reduced set into the NN.

In addition to combinatorial approaches, hierarchical approaches can also be used to increase scrutinization and consequently source separation. A study by Bayliss et al. (2023), used hierarchical ML classifier to determine the geographical origin of different isolates, on a variety of different spatial levels (such as continent, region, and country). A similar approach could be taken in source attribution, attributing on different strata, such as on the species, population and subpopulation levels. Indeed, a hierarchical random forest has previously been used to make a preselection of features relevant to specific countries to enhance correct source attribution predictions (Duarte et al., 2020). As discussed in the metadata section, metadata can be used to split data on. This may help alleviate problems regarding the separation of data wherein the cluster coherence of certain classes is low.

Stratification may also work to subdivide pathogens, which have differently behaving strains. Previous studies have noted that there exists host restricted and broad host range *Salmonella enterica* serovar Typhimurium groups (Rabsch et al., 2002; Parsons et al., 2013; Kingsley et al., 2013). Lupolova et al. (2019) attempted to separate the data of *S. enterica* serovar Typhimurium based on a classification of generalists and specialists, which was based on host range. Such a separation, may be applicable to any hierarchical ML approach.

### 3.2 Metadata

The presence of strains with a wide host range within different hosts often makes the separation of data difficult. This makes a definite assignment precarious, and consequently probability-based assignments are the standard choice. Nevertheless, source separation may be reached through the inclusion of additional dimensionality to separate data on. This may be done through the addition of filters, features, weights, constraints, etc.

Spatial data, for instance, may enhance within source cluster variance. This is due to the potentiality of geographically separated populations to evolve independently from one another despite similarities in niches (Lupolova et al., 2019). Consequently, the geographical scope of sampling reflects the capture of finer or less-distinct data patterns. Nevertheless, if factors like travel and trade have not been accounted for, they can obfuscate these patterns (Smid et al., 2013; Bayliss et al., 2023).

A complicating factor that should also be considered is the difference in the ranging behavior of hosts, which may influence the impact of a spatial dimension on source attribution between sources (Griekspoor et al., 2013). In this manner, genomic diversity within the same genus, species and between strains may be enhanced. A larger ranging habitat means a less distinct geographical pattern.

The incorporation of a temporal dimension may help separate pathogen sources as well. After all, fast genomic changes can cause changes within pathogen populations on a short temporal scale (Smid et al., 2013), and lead to a drift within a between strains, genus and species on a spatial–temporal scale. The assignment of a new isolate to a previously categorized source may therefore simply be convergent evolution, or incidental similarities. To prevent the introduction of temporal biases, the temporal aspect should not be ignored.

As such, periodic surveillance is a necessity to guarantee source and patient isolate completeness. However, surveillance is time-consuming and costly. Therefore, studies need to be performed to ascertain the maximum time past between data acquisitions for relevant sources, which will allow deriving ancestry of retrieved patient isolates. This may differ between pathogens based on how conserved their genome is. Moreover, even between populations or subpopulations of pathogens this may differ radically.

There also exist seasonal patterns of disease and source composition. In part this may be due to the population dynamics of pathogens, changes in the presence and ranging behavior of animals, and shifts in human behavior throughout the year. Bayliss et al. (2023) noted that the infection rates of *Salmonella Enteritidis* were highly seasonal in both Europe and Asia, with the highest rates of infection occurring during summer. Other papers corroborate the same link between seasonality or climate and infections (Lin et al., 2016; Dhimal et al., 2022; John et al., 2022).

With more complete and larger volumes of captured data different methods of separating data, for example through metadata will more pertinent. For cgMLST, this directly impacts the size of the core genome, shrinking it as the dataset grows. A way to address this is by including a selected set of representative isolates. A study from 2021 by Abram et al. (2021), stratified *E. coli* into different phylogroups. Introducing a reference genome in such a manner, from foodborne pathogens, will make identification and genome assembly more robust, and the link between ecological differences found in pathogroups may be reflected in the makeup of the phylogroups. Alternatively, pangenomes could potentially be used to function as representative isolates (Svahn et al., 2023).

Another potential problem to be kept in mind is the transient presence of a pathogen within a host, which may lead to wrong assignment of the source (Dearlove et al., 2016). For example, an animal may be a short layover before switching again, or the pathogen might have very recently infected the host species. Since the direction of infection is unknown, this transient presence is a complicating factor. In part, rapid host switching allows for the genetic exchange between strains on a more frequent basis, thus a less clear separation between sources and strains, which further complicates assignment (Dearlove et al., 2016).

Despite this, the poor characterization of metadata within most data, it could serve as a strong means of filtering, separating or clustering data on.

### 3.3 Metagenomics

Metagenomics offers a culture-independent approach to source attribution, allowing for the potential characterization of all pathogens within an environment through sequencing. Additionally, metagenomics gives an indication of the population structure of the sequenced environment (e.g., gut, sewage, etc.) (Tyson et al., 2004).

Regarding source attribution, metagenomics is a barely explored direction. It is notable that characterization of specific pathogens is difficult; however, the community layout can be captured (Ko et al., 2022). This allows for the exploration of a wide variety of samples, e.g., wastewater surveillance, for the attribution of AMR from diverse sources. Therefore, metagenomics can be used to determine temporal and spatial shifts in population makeup. In addition, a study by Duarte et al. (2020), has performed comparisons of metagenomes of humans and sources, through specific markers, thus allowing source attribution based on community level compositions. Specifically, they used the antimicrobial resistance (AMR) composition of different sources to infer the likely source of human metagenomic samples. Based on the assumption that the AMR abundances are determined in part by HGT and are specific to the fecal resistome, they were able to attribute metagenomic samples to sources.

However, metagenomic approaches disregard individual pathogens and particular contributions of specific pathogens within samples. In addition, pathogens must be present in high enough numbers to be able to be detected through metagenomics (Escobar-Zepeda et al., 2016). Since the community composition where disease is manifested can differ radically from the community composition of asymptomatic or unaffected hosts, there exists an uncertainty whether a pathogen is simply absent or present in far too low numbers within the host. This is further aggravated by lower levels of resolution for pathogens in metagenomic samples as opposed to sequenced isolates. As part of surveillance, source attribution serves a role in partially characterizing the pathogenic entity; therefore, genomics approaches might be preferred.

### 3.4 Network approaches

Network approaches are a feasible option for source attribution, based on the conceptional consideration of a relation existing between the human and source isolates. This forms a bipartite system, which is represented through the genetic relatedness being shown as a weighted link. If two genomes are derived from the same source, the expectation would be a smaller distance value. As such, cluster coherence of reservoirs, together with the driving forces of parameters in the formation of clusters, can be evaluated. In this manner, networks of communities can be retrieved (Wainaina et al., 2022). To date, not many studies have been performed using network approaches. However, Merlotti et al. (2020) and Wainaina et al. (2022) performed preliminary investigations on *Salmonella enterica* serovar Typhimurium and *Campylobacter* spp. respectively. Both papers were able to attribute sources to human pathogen isolates. Nevertheless, the coherence of the clusters was markedly less in *Campylobacter*, which might be due to the high genome diversity as compared to *Salmonella Typhimurium*.

One of the benefits of network approaches is that clustering is not necessarily bound to a particular source, rather to clusters, which may contain different sources (links are represented as pairwise distances). This is meant to reflect the distribution of particular pathogen populations through the sources. However, it may just as well indicate problematics concerning the separation of sources. The clustering of human isolates with clusters composed of other sources is therefore portrayed in probabilities reflecting this uncertainty.

On the other hand, a potential problem is the under- or over-representation of clusters. This is especially true when dealing with high interspecies genetic diversity, unclear source separation, or uncertainties within the metadata.

## 4 Hazard characterization

### 4.1 Current approaches and breadth

Besides cluster and outbreak detection, the characterization of strains is a crucial aspect in infectious disease surveillance in order to assess the risk of strains, for individual patients or public health, encountered during surveillance and to keep track of their circulation. For foodborne pathogens, this mainly involves using genes or genomic elements to investigate specific antimicrobial resistance profiles and virulence profiles affecting humans. It may also involve characteristics involved in transmissibility and immune evasion. When using WGS for surveillance, hazard characterization can be conducted relatively easily once the targets are known. Standard approaches to hazard characterization of pathogens using WGS data concerns the evaluation of sequence data. A variety of tools exist to characterize virulence genes (Ren et al., 2017; Malberg Tetzschner et al., 2020), AMR markers (Bortolaia et al., 2020; Feldgarden et al., 2019; Zankari et al., 2017; Alcock et al., 2020), and other genetic factors (Carattoli and Hasman, 2020; Siguier et al., 2006; Camacho et al., 2009; Yoon et al., 2014). Some tools are tailored toward specific species. In addition, WGS holds promise in the point-of-care conditions of patients by alleviating the dependency on culture-based antibiotic tests. WGS would resolve complications regarding comparability between different labs and samples, reanalysis and storage (Verschuuren et al., 2022). Furthermore, if sequencing can be uncoupled from the need to cultivate the pathogen, through for example metagenomics, then the time required for sequencing could be measurable less as compared to culture-based antibiotics tests (Deurenberg et al., 2017).

However, identifying useful targets is a difficult process and may be performed with a plethora of techniques. Difficulties are exacerbated by a variety of problems. Firstly, a majority of proteins is uncharacterized or lack a close functional homolog with a sequence identity above 60%. The less the isolate sequences share in similarity with the nearest characterized sequence, the more uncertain the derived function becomes. Rapid evolution leading to high sequence divergence may thus obscure homology. Alternatively, the gene may encode an unknown function or may be a novel gene. In these cases, a variety of tools may help determine gene function. By leveraging information about amino acid interactions to determine structural information of the proteins, a variety of tools can give an indication of function (Yang et al., 2023; van Kempen et al., 2024; Jumper et al., 2021; Jin et al., 2021). Nevertheless, high similarity does not confirm similar functions. This necessitates laboratory experiments, where bioinformatics tools are not able to validate the function of a protein. Secondly, the genomic data are often uncoupled from epidemiological case/control data, which would allow for further evaluation of the hazard. Thirdly, limitations in sequencing technologies, uncertainty in regard to inferred function, biases associated with applied methodology, and problems specific to these methodologies exacerbate the difficulty of identifying targets. Methods can be rooted in genomics, such as Genome-wide association studies (GWAS) (Dutilh et al., 2013), ML approaches (Wheeler et al., 2018), and approaches involving metagenomics (Duarte et al., 2020). All in all patterns of genes associated with risk, regions of the genomes, or specific mutations can be uncovered relevant for hazard characterization.

### 4.2 Approaches to characterize new targets

#### 4.2.1 Genome-wide association studies

Genome-wide association studies (GWAS) refer to studies, which look at genetic variants within a genome-wide context to discover gene (kmer/SNP) variants related to a particular phenotypic trait. To find variants, a “test” and “control” group are compared to spot genetic variation associated with the trait under scrutiny. Shortly, a reference genome is used to align the genome. Then a cut-off can be calculated from the allele/genetic frequencies above which an association between genotype and phenotype is highly likely (Uffelmann et al., 2021). Alternatively, reference free GWAS may be performed with associations made between k-mers (Mehrab et al., 2020). Nevertheless, at the core of GWAS is the hypothesis stating a plethora of variants exist with a low effect that together can form complex inheritance patterns, causing genetic and phenotypic heterogeneity (Power et al., 2017).

In order to make strong inferences about associations with GWAS, a substantial amount of data is required. Consequently, the epoch of next-generation sequencing (especially WGS), with its’ multitudes of generated data, has allowed GWAS to be considered as a tool in bacterial genomics (Dutilh et al., 2013). In fact, many studies have been performed relating to hazard characterization (Chaguza et al., 2022; Sephton-Clark et al., 2022). Even within the context of foodborne disease, the number of studies performed is increasing (Tiwari et al., 2023; Epping et al., 2021; Buchanan et al., 2017).

However, there is still a diverse array of problems with regard to applying GWAS to microbial genomics. Some are general to GWAS, whereas others are rooted in the behavior and character of bacterial pathogen populations. Specific to GWAS, sample size and effect sizes (and distribution of effect sizes) are the dominant drivers of the power of GWAS associations (Saber and Shapiro, 2020). The effect sizes within bacterial genomes tend to grow, since these often resulted from recent selection (Duchen et al., 2023). Other relevant bacterial specific effects causing complications are linkage disequilibriums interrupted by homologous recombinations and strong population structures resulting from clonal expansion. The prevalence of the linkage disequilibrium in bacterial genomes may cause wrong associations to effects (Saber and Shapiro, 2020). Meanwhile, clonal expansion can cause associations based on ancestry (Saber and Shapiro, 2020; Power et al., 2017).

In a study by Saber and Shapiro (2020), various approaches to correct for population structure and linkage disequilibrium were evaluated. The study modeled evolutionary parameters to simulate various bacterial behaviors, varying sample size, causal variant effect size and linkage disequilibrium, to find the optimal parameters and approach. They found that larger samples sizes and effect sizes perform best. Nevertheless, they do not account for variable mutational rates, which are largely influenced by pathogen behavior.

Disease severity may not be linked completely to the pathogen, because also varieties in hosts play a part in the pathogen-disease interplay, such as susceptibility, immune reactions, genome variations in these hosts etc. (Power et al., 2017). In addition, subtypes may differ in phenotypes, thus correct classification of lineages may proof important to link effect to SNPs or k-mers (Power et al., 2017). Moreover, a correct choice of control subject needs to be made to circumvent selection biases predicated on environmental exposure (Duchen et al., 2023). These complications may hinder GWAS. To the extent of correcting for these various problems encountered during bacterial GWAS there exist a variety of tools. TreeWAS (Collins and Didelot, 2018) and Hogwash (Saund and Snitkin, 2020) exist to correct for the population structure, whereas PowerBacGWAS (Coll et al., 2022) helps figure out the required sample size to be able to infer associations.

At present, the availability of genomes with phenotypic information known is much smaller compared to human GWAS. Nevertheless, many studies have found success, perhaps because of the large effect size of microbes. With time, the number of available sequences is sure to grow, thus increasing the statistical power of GWAS; however, the effects of heterogeneity within a dataset must be categorized in order to be able to say with certainty GWAS is applied correctly and discriminatingly (Power et al., 2017).

#### 4.2.2 Machine learning approaches for hazard characterization

Aforementioned ML techniques, mentioned in the source attribution section, find a variety of utilities within the field of hazard characterization, such as in the prediction of antibiotic resistance (Moradigaravand et al., 2018). Gathered metadata may be used as class data, in combination with available nucleotide/allele schemes to predict disease severity or pathogenicity. In theory, the algorithm should train on patterns of occurring allele/nucleotide profiles associated with a particular class. The extraction of relevant features responsible for a particular class assignment could then be used to find genes of interest related to, e.g., disease severity.

Previous studies have already applied ML models to these ends. One study sought to differentiate pathogenic from non-pathogenic STEC (Im et al., 2021), based on the assumption that clinical isolates and those from known sources, such as cows, are pathogenic, whereas environmental isolates are not. Whereas, another study was able to apply RF to identify patterns of adaptation associated with disease caused by *Salmonella enterica* (Wheeler et al., 2018).

Similarly, genomic differences can be recognized through methodologies such as convolutional neural networks (Ciresan et al., 2011; Quang and Xie, 2016), which can be trained to recognize differences within DNA sequences. For example, pathogenicity islands, transposons, and mobile genetic elements differ in genetic makeup from the surrounding sequences and as such can be differentiated (Lu and Leong, 2016). Nevertheless, long-read sequencing may be able to resolve the full genetic structure of the genome from the different sequence reads.

As such, many forays into ML applications for hazard characterization are feasible and have proven fruitful (Wheeler et al., 2018; Im et al., 2021). However, comparisons between different methodologies ought to be made for providing the best predictions. Therefore, different ML approaches should be benchmarked in order to uncover the relevance and applicability solitarily or in combination with other approaches.

#### 4.2.3 Large language models

Large language models (LLMs) have seen popular implementation in applications such as ChatGPT and for the translation of hitherto unknown ancient languages (Luo et al., 2019); however, their implications for the field of biology are also apparent. For example, LLMs could be used for the translation of DNA to proteins, but more interestingly for predicting evolutionary changes within DNA.

A study from 2022 by Zvyagin et al. (2022) sought to uncover the evolutionary dynamics of SARS-CoV-2 by training a LLM on prokaryotic gene sequences (110 million in total) and fine-tuning these based on SARS-CoV-2 sequences (1.5 million genomes in total).

However, the implementation of such a model in surveillance would require access to enormous volumes of data, which are currently unavailable for foodborne bacteria. Nevertheless, since we are dealing with increasingly larger amounts of quantitative data, the potential role of LLMs in surveillance should be kept in the back of our minds.

#### 4.2.4 Network approaches

Network approaches have been used to predict pathogen evolution in the context of surveillance (Cliff et al., 2020; Chang et al., 2023). For example, to the extent of modeling genetic diversity in comparison to the evolutionary niche and the relation to disease outbreaks (Chang et al., 2023). Another example is about mapping the prevalence and spread of pathogens together with evolutionary pathways to examine possible trends across the spatial–temporal axis (Cliff et al., 2020). In this way, these models provide us an understanding of the evolutionary dynamics, which define surveillance practices. Nonetheless, many avenues of research are still left. The dynamics of these networks, their structures, and the implications of changes within these networks need to be evaluated. Furthermore, the utility of network approaches for the modeling of AMR, pathogenicity, and host switching could be evaluated.

#### 4.2.5 Metagenomics and other-omics approaches

Since the different -omics approaches strictly fall outside the confines of this review, we will only briefly touch upon the utility of -omics approaches. These approaches have proven especially relevant in the field of microbial risk assessment, where the phenotypic and genotypic response may be very different and the association of genes to a phenotypic response is not always understood. AMR genes have been exhaustively described in literature, however, the phenotypic response for other virulence-related genes is often not as clear (Leekitcharoenphon et al., 2021). In fact, using -omics approaches concomitantly with genomics may help provide links between the phenotypic response and presence absence of genes or SNPs. For instance, transcriptomics and proteomics can be employed for assessing virulence, pathogenicity, and AMR (for an in-depth review, see Haddad et al., 2018 and Bergholz et al., 2014). Based on the relative expressions of RNAs and proteins, more insight can be gained into differences in disease severity, immune system evasion, AMR and other phenotypic traits. For example, a mutation may cause weaker binding of polymerases, and consequently the production of less proteins, which for instance may be related to pathogenicity. Information about how pathways are modulated and regulated, and how this may be tweaked to our benefit can thus be obtained (Bergholz et al., 2014). The utility of metabolomics in the field of microbial risk assessment is shown through the ability to capture AMR profiles (Ma et al., 2021). Nevertheless, it must be noted that for many -omics approaches the availability of large datasets is limited.

Metagenomics, likewise, can be exploited for hazard characterization. For example, the entirety of the pathogen reservoir captured within a sample can be observed using metagenomics. Characterization of the AMR, virulence genes, pathogenic genes, stress-related genes, or gene variants (Duarte et al., 2020; Jaudou et al., 2022; Díaz-Palafox et al., 2023), for instance may allow us to estimate associated risk. HGT between different pathogen subtypes, may radically change the disease expression of said pathogen. After all, the effect size of genetic changes in microbes is rather large. Therefore, metagenomics allows for population scale hazard characterization of various factors implicated herein. In addition, through co-occurrence or exclusion of species in presence of one another, the risk at a community level can be assessed (Alessandria et al., 2016). Thereby, metagenomics may reveal a core community within an environment as well (Chaillou et al., 2015).

However, disentangling sequences of individual isolates from the population is a complex and time-consuming task. A second problem related to the need for pathogens to be present in high enough numbers to be able to be detected through metagenomics (Escobar-Zepeda et al., 2016). Therefore, where characterization of distinct strains, species or subspecies is concerned WGS is preferred (Table 2).

**Table 2:** Comparison analysis approaches in clustering/outbreak detection, source attribution, and hazard characterization.

	Clustering & Outbreak detection	Source Attribution	Hazard Characterization
Threshold	Easily applicable based on historical data, however do not reflect differences between isolates/strains/species.	Unsupervised machine learning could be applied, herein the clusters represent the source groups, and however, uncertainty about the number of sources exists. Similarly supervised machine learning can be applied where the predictions are the different sources.	Predict disease severity, pathogenicity, which may then be used to extract relevant features. In addition, machine learning may be used to recognize different genetical elements.
Modeling & Dynamic thresholds	Require metadata, but allow dynamic changes in parameters to more closely reflect different cut-offs between clusters.	Genetic relatedness shown as weighted links. Closely linked isolates are expected to be derived from the same cluster. In particular clustering is not necessarily bound to a particular source, but clusters containing different sources reflecting source uncertainty.	Predict pathogen evolution in context of surveillance.
Machine learning	Unsupervised machine learning can be used to clustering, however requires a pre-determined number of clusters, therefore prior knowledge is required.	Allows source attribution based on community level composition, however, individual isolates are often disregarded as are particular contributions of specific pathogens within the samples.	Estimate associated risk on a community scale level. Thereby, metagenomics may reveal a core community within an environment as well.
Network analyses	Network with connectivity between isolates showing clustering.		Looks at genetic variants within a genome-wide context to discover gene variants related to a particular phenotypic trait.
Metagenomics			Not enough data yet, but could in the future be used to predict evolutionary changes.
GWAS			Together with genomics approaches may provide a link between phenotypic and genotypic response.
LLM			Future potential for predicting evolutionary changes in DNA sequences.
Other—omics approaches			In combination with WGS allow for linking of phenotype and genotype response.

## 5 Conclusion

Improvements in sequencing quality, availability, speed, and costs results in an increased presence of genomics in infectious disease applications, such as surveillance, source attribution, and hazard characterization. We provided an overview of existing and new approaches to use for WGS data. Highlighting the benefits, downsides, and current hurdles to overcome in order to improve hazard characterization, source attribution and clustering/outbreak detection in the future. Among the applications, we especially highlighted ML and network analysis as non-traditional approaches for cluster definition, determining the relative importance of sources and reservoirs, and to identify pathogen traits associated with increased risk. It should be stressed that no universal analysis or approach exists and the best method to apply depends on the goal and data availability.

Although these methods can relatively easily be applied, an overarching challenge is the inference and biological/epidemiological interpretation of these large amounts of high resolution data. Maybe more than ever context is crucial. Especially understanding the genomic diversity of the pathogen population studied is pivotal for assessing cluster identification in surveillance, performing source attribution studies and conducting hazard characterization of isolates. Understanding the context in terms of diversity allows to assess the level of representativeness of the dataset, which in turn defines the level of certainty associated with defining clusters, sources and risks. This also marks the importance of metadata (clinical, epidemiological, and biological) when using genomics for public health purposes. Here metadata provides a manner to classify relatedness and diversity based on additional data, such as spatial–temporality and disease outcome.

The quantitative increase in sequencing data may yet support more elaborate and data “heavy” analyses. Concomitantly, data are increasingly an amalgamation of various points and places in time. Therefore, thought needs to be put into the spatial–temporality aspect of the data. Complete captures of all sources, temporal effects on pathogen behavior, the spatial spread of clusters, transmission pathways, temporal effects on spatiality, and the temporal effect on cluster cohesion present uncertainties in regard to larger scale research combining data on a larger spatial-temporal axis. Continued and consistent recurrent surveillance may help answer these questions.

In the same vein, understanding of population dynamics can be gleaned from the use of metagenomics, but the method is largely restricted to a community level view. The ability to disentangle individual strains from the community composition would be favorable.

Meanwhile, larger datasets require more due consideration for the choice of reference to construct the typing scheme on. The typing scheme and resultingly resolution may be limited by the sequencing approach applied and the volume of data. Improvement of long-read sequencing may help capture previously uncaptured regions, but too many details may cause over-discrimination where unnecessary. In certain cases, a more concise and inclusive dataset may take precedence over a large more generative dataset. Again, context needs to be considered for both strains and the dataset being inquired.

Within the right context hazard characterization, source attribution and clustering for outbreak detection can be utilized to great effect, but are still hampered by methodological considerations. The designation of a cluster requires a cut-off, which in turn require inferring this cut-off. Similarly, probabilistic methods do not give a definite assignment and uncertainty regarding pathogen behavior or population may hinder correct assignment. GWAS is subject to uncertainty about lineages, linkage of effects to genes and other biases. Whereas, inference based on a database may be subject to bias. Therefore, different approaches (in combination) need to be benchmarked not for their general application, but rather for their specific use-cases.

Finally, the approaches, methods and studies discussed within this paper are all limited by and adapt to the scope of the information available. Typing method and approach to analysis should be chosen in accordance. However, external information regarding the pathogen studied should be taken into consideration. Pathogen diversity in terms of behavior and clustering can heavily bias the resulting research. In addition, the composition of the data needs to be considered as well. Resultingly, different methods should be benchmarked to uncover the relevance and applicability for pathogen strains through space and time. In addition, resolving uncertainties regarding sources, spread and evolutionary dynamics of pathogen strains will be key in understanding the drivers for hazard characterization, source assignment and cluster formation. These drivers need to be put within their biological/epidemiological imperative in order for the methodological approaches to make sense. Continued and more ubiquitous surveillance together with a move toward long-read sequencing may therefore be key in resolving these issues. However, caution needs to be taken regarding the growing amounts of data, such that due to the numerous trees we do not lose sight of the forest.

## References

[ref1] Abram K. Udaondo Z. Bleker C. Wanchai V. Wassenaar T. M. Robeson M. S. 2nd. (2021). Mash-based analyses of *Escherichia coli* genomes reveal 14 distinct phylogroups. Commun. Biol. 4:117. doi: 10.1038/s42003-020-01626-5 33500552 PMC7838162

[ref2] Aladhadh M. (2023). A review of modern methods for the detection of foodborne pathogens. Microorganisms 11:1111. doi: 10.3390/microorganisms11051111, PMID: 37317085 PMC10221273

[ref3] Alcock B. P. Raphenya A. R. Lau T. T. Y. Tsang K. K. Bouchard M. Edalatmand A.. (2020). Card 2020: antibiotic resistome surveillance with the comprehensive antibiotic resistance database. Nucleic Acids Res. 48, D517–D525. doi: 10.1093/nar/gkz935, PMID: 31665441 PMC7145624

[ref4] Alessandria V. Ferrocino I. De Filippis F. Fontana M. Rantsiou K. Ercolini D.. (2016). Microbiota of an Italian grana-like cheese during manufacture and ripening, unraveled by 16S rRNA-based approaches. Appl. Environ. Microbiol. 82, 3988–3995. doi: 10.1128/AEM.00999-16, PMID: 27107125 PMC4907185

[ref6] Amarasinghe S. L. Su S. Dong X. Zappia L. Ritchie M. E. Gouil Q. (2020). Opportunities and challenges in long-read sequencing data analysis. Genome Biol. 21:30. doi: 10.1186/s13059-020-1935-5 32033565 PMC7006217

[ref7] Arning N. Sheppard S. K. Bayliss S. Clifton D. A. Wilson D. J. (2021). Machine learning to predict the source of campylobacteriosis using whole genome data. PLoS Genet. 17:e1009436. doi: 10.1371/journal.pgen.1009436, PMID: 34662334 PMC8553134

[ref8] Arnold B. J. Huang I. T. Hanage W. P. (2022). Horizontal gene transfer and adaptive evolution in bacteria. Nat. Rev. Microbiol. 20, 206–218. doi: 10.1038/s41579-021-00650-4, PMID: 34773098

[ref9] Atxaerandio-Landa A. Arrieta-Gisasola A. Laorden L. Bikandi J. Garaizar J. Martinez-Malaxetxebarria I.. (2022). A practical bioinformatics workflow for routine analysis of bacterial WGS data. Microorganisms 10:2364. doi: 10.3390/microorganisms10122364, PMID: 36557617 PMC9781918

[ref10] Barrick J. E. Lenski R. E. (2013). Genome dynamics during experimental evolution. Nat. Rev. Genet. 14, 827–839. doi: 10.1038/nrg3564, PMID: 24166031 PMC4239992

[ref11] Bayliss S. C. Locke R. K. Jenkins C. Chattaway M. A. Dallman T. J. Cowley L. A. (2023). Rapid geographical source attribution of *Salmonella enterica* serovar Enteritidis genomes using hierarchical machine learning. eLife 12:e84167. doi: 10.7554/eLife.84167, PMID: 37042517 PMC10147375

[ref12] Bergholz T. M. Moreno Switt A. I. Wiedmann M. (2014). Omics approaches in food safety: fulfilling the promise? Trends Microbiol. 22, 275–281. doi: 10.1016/j.tim.2014.01.006, PMID: 24572764 PMC4016976

[ref13] Bortolaia V. Kaas R. S. Ruppe E. Roberts M. C. Schwarz S. Cattoir V.. (2020). ResFinder 4.0 for predictions of phenotypes from genotypes. J. Antimicrob. Chemother. 75, 3491–3500. doi: 10.1093/jac/dkaa345, PMID: 32780112 PMC7662176

[ref14] Brinch M. L. Hald T. Wainaina L. Merlotti A. Remondini D. Henri C.. (2023). Comparison of source attribution methodologies for human Campylobacteriosis. Pathogens 12:786. doi: 10.3390/pathogens12060786, PMID: 37375476 PMC10303420

[ref15] Brown E. Dessai U. Mcgarry S. Gerner-Smidt P. (2019). Use of whole-genome sequencing for food safety and public health in the United States. Foodborne Pathog. Dis. 16, 441–450. doi: 10.1089/fpd.2019.2662, PMID: 31194586 PMC6653787

[ref16] Buchanan C. J. Webb A. L. Mutschall S. K. Kruczkiewicz P. Barker D. O. R. Hetman B. M.. (2017). A genome-wide association study to identify diagnostic markers for human pathogenic *Campylobacter jejuni* strains. Front. Microbiol. 8:1224. doi: 10.3389/fmicb.2017.01224 28713351 PMC5492696

[ref17] Camacho C. Coulouris G. Avagyan V. Ma N. Papadopoulos J. Bealer K.. (2009). BLAST+: architecture and applications. BMC Bioinformatics 10:421. doi: 10.1186/1471-2105-10-421 20003500 PMC2803857

[ref18] Carattoli A. Hasman H. (2020). PlasmidFinder and in silico pMLST: identification and typing of plasmid replicons in whole-genome sequencing (WGS). Methods Mol. Biol. 2075, 285–294. doi: 10.1007/978-1-4939-9877-7_20 31584170

[ref19] Chaguza C. Smith J. T. Bruce S. A. Gibson R. Martin I. W. Andam C. P. (2022). Prophage-encoded immune evasion factors are critical for *Staphylococcus aureus* host infection, switching, and adaptation. Cell Genom. 2:100194. doi: 10.1016/j.xgen.2022.100194, PMID: 36465278 PMC9718559

[ref20] Chaillou S. Chaulot-Talmon A. Caekebeke H. Cardinal M. Christieans S. Denis C.. (2015). Origin and ecological selection of core and food-specific bacterial communities associated with meat and seafood spoilage. ISME J. 9, 1105–1118. doi: 10.1038/ismej.2014.202, PMID: 25333463 PMC4409155

[ref21] Chang S. L. Suster C. J. E. Rockett R. J. Svahn A. J. Cliff O. M. Arnott A.. (2023). Genome entropy and network centrality contrast exploration and exploitation in evolution of foodborne pathogens. Phys. Biol. 20:046006. doi: 10.1088/1478-3975/acd899, PMID: 37224820

[ref22] Ciresan D. Meier U. Masci J. Gambardella L. M. Schmidhuber J. (2011). Flexible, high performance convolutional neural networks for image classification.

[ref23] Cliff O. M. Mclean N. Sintchenko V. Fair K. M. Sorrell T. C. Kauffman S.. (2020). Inferring evolutionary pathways and directed genotype networks of foodborne pathogens. PLoS Comput. Biol. 16:e1008401. doi: 10.1371/journal.pcbi.1008401, PMID: 33125373 PMC7657559

[ref24] Coipan C. E. Dallman T. J. Brown D. Hartman H. Van Der Voort M. Van Den Berg R. R.. (2020). Concordance of SNP- and allele-based typing workflows in the context of a large-scale international *Salmonella Enteritidis* outbreak investigation. Microb. Genom. 6:e000318. doi: 10.1099/mgen.0.000318, PMID: 32101514 PMC7200063

[ref25] Coipan C. E. Friesema I. H. Van Den Beld M. J. C. Bosch T. Schlager S. Van Der Voort M.. (2022). Sporadic occurrence of Enteroaggregative Shiga toxin-producing *Escherichia coli* O104:H4 similar to 2011 outbreak Strain. Emerg. Infect. Dis. 28, 1890–1894. doi: 10.3201/eid2809.220037, PMID: 35997633 PMC9423916

[ref26] Coll F. Gouliouris T. Bruchmann S. Phelan J. Raven K. E. Clark T. G.. (2022). PowerBacGWAS: a computational pipeline to perform power calculations for bacterial genome-wide association studies. Commun. Biol. 5:266. doi: 10.1038/s42003-022-03194-2 35338232 PMC8956664

[ref27] Coll F. Raven K. E. Knight G. M. Blane B. Harrison E. M. Leek D.. (2020). Definition of a genetic relatedness cutoff to exclude recent transmission of meticillin-resistant *Staphylococcus aureus*: a genomic epidemiology analysis. Lancet Microb. 1, e328–e335. doi: 10.1016/S2666-5247(20)30149-X, PMID: 33313577 PMC7721685

[ref28] Collins C. Didelot X. (2018). A phylogenetic method to perform genome-wide association studies in microbes that accounts for population structure and recombination. PLoS Comput. Biol. 14:e1005958. doi: 10.1371/journal.pcbi.1005958, PMID: 29401456 PMC5814097

[ref29] Compeau P. E. Pevzner P. A. Tesler G. (2011). How to apply de Bruijn graphs to genome assembly. Nat. Biotechnol. 29, 987–991. doi: 10.1038/nbt.2023, PMID: 22068540 PMC5531759

[ref30] Cori A. Nouvellet P. Garske T. Bourhy H. Nakouné E. Jombart T. (2018). A graph-based evidence synthesis approach to detecting outbreak clusters: an application to dog rabies. PLoS Comput. Biol. 14:e1006554. doi: 10.1371/journal.pcbi.1006554, PMID: 30557340 PMC6312344

[ref31] Dallman T. J. Byrne L. Ashton P. M. Cowley L. A. Perry N. T. Adak G.. (2015). Whole-genome sequencing for national surveillance of Shiga toxin-producing *Escherichia coli* O157. Clin. Infect. Dis. 61, 305–312. doi: 10.1093/cid/civ318, PMID: 25888672 PMC4542925

[ref32] Dallman T. J. Greig D. R. Gharbia S. E. Jenkins C. (2021). Phylogenetic structure of Shiga toxin-producing *Escherichia coli* O157:H7 from sub-lineage to SNPs. Microb. Genom. 7:mgen000544. doi: 10.1099/mgen.0.000544, PMID: 33720818 PMC8190602

[ref33] Dearlove B. L. Cody A. J. Pascoe B. Méric G. Wilson D. J. Sheppard S. K. (2016). Rapid host switching in generalist Campylobacter strains erodes the signal for tracing human infections. ISME J. 10, 721–729. doi: 10.1038/ismej.2015.149, PMID: 26305157 PMC4677457

[ref34] Den Bakker H. C. Allard M. W. Bopp D. Brown E. W. Fontana J. Iqbal Z.. (2014). Rapid whole-genome sequencing for surveillance of *Salmonella enterica* serovar enteritidis. Emerg. Infect. Dis. 20, 1306–1314. doi: 10.3201/eid2008.131399, PMID: 25062035 PMC4111163

[ref35] Deurenberg R. H. Bathoorn E. Chlebowicz M. A. Couto N. Ferdous M. Garcia-Cobos S.. (2017). Application of next generation sequencing in clinical microbiology and infection prevention. J. Biotechnol. 243, 16–24. doi: 10.1016/j.jbiotec.2016.12.022, PMID: 28042011

[ref36] Dhimal M. Bhandari D. Karki K. B. Shrestha S. L. Khanal M. Shrestha R. R. P.. (2022). Effects of climatic factors on diarrheal diseases among children below 5 years of age at national and subnational levels in Nepal: an ecological study. Int. J. Environ. Res. Public Health 19:6138. doi: 10.3390/ijerph19106138, PMID: 35627674 PMC9140521

[ref37] Díaz-Palafox G. Tamayo-Ordoñez Y. J. Bello-López J. M. Ayil-Gutiérrez B. A. Rodríguez-Garza M. M. Antonio Rodríguez-De La Garza J.. (2023). Regulation transcriptional of antibiotic resistance genes (ARGs) in Bacteria isolated from WWTP. Curr. Microbiol. 80:338. doi: 10.1007/s00284-023-03449-z, PMID: 37672120 PMC10482803

[ref39] Duarte A. S. R. Röder T. Van Gompel L. Petersen T. N. Hansen R. B. Hansen I. M.. (2020). Metagenomics-based approach to source-attribution of antimicrobial resistance determinants—identification of reservoir Resistome signatures. Front. Microbiol. 11:601407. doi: 10.3389/fmicb.2020.601407, PMID: 33519742 PMC7843941

[ref40] Duchen D. Vergara C. Thio C. L. Kundu P. Chatterjee N. Thomas D. L.. (2023). Pathogen exposure misclassification can bias association signals in GWAS of infectious diseases when using population-based common control subjects. Am. J. Hum. Genet. 110, 336–348. doi: 10.1016/j.ajhg.2022.12.013, PMID: 36649706 PMC9943744

[ref41] Dutilh B. E. Backus L. Edwards R. A. Wels M. Bayjanov J. R. Van Hijum S. A. (2013). Explaining microbial phenotypes on a genomic scale: GWAS for microbes. Brief. Funct. Genomics 12, 366–380. doi: 10.1093/bfgp/elt008, PMID: 23625995 PMC3743258

[ref42] Duval A. Opatowski L. Brisse S. (2023). Defining genomic epidemiology thresholds for common-source bacterial outbreaks: a modelling study. Lancet Microb. 4, e349–e357. doi: 10.1016/S2666-5247(22)00380-9, PMID: 37003286 PMC10156608

[ref43] Epping L. Walther B. Piro R. M. Knüver M.-T. Huber C. Thürmer A.. (2021). Genome-wide insights into population structure and host specificity of *Campylobacter jejuni*. Sci. Rep. 11:10358. doi: 10.1038/s41598-021-89683-6 33990625 PMC8121833

[ref44] Escobar-Zepeda A. Sanchez-Flores A. Quirasco Baruch M. (2016). Metagenomic analysis of a Mexican ripened cheese reveals a unique complex microbiota. Food Microbiol. 57, 116–127. doi: 10.1016/j.fm.2016.02.004, PMID: 27052710

[ref45] Feldgarden M. Brover V. Haft D. H. Prasad A. B. Slotta D. J. Tolstoy I.. (2019). Validating the AMRFinder tool and resistance gene database by using antimicrobial resistance genotype-phenotype correlations in a collection of isolates. Antimicrob. Agents Chemother. 63:e00483-19. doi: 10.1128/AAC.00483-19, PMID: 31427293 PMC6811410

[ref46] Franz E. Gras L. M. Dallman T. (2016). Significance of whole genome sequencing for surveillance, source attribution and microbial risk assessment of foodborne pathogens. Curr. Opin. Food Sci. 8, 74–79. doi: 10.1016/j.cofs.2016.04.004

[ref47] Fu Y. Mikanatha N. M. Lorch J. M. Blehert D. S. Berlowski-Zier B. Whitehouse C. A.. (2022). *Salmonella enterica* Serovar typhimurium isolates from wild birds in the United States represent distinct lineages defined by bird type. Appl. Environ. Microbiol. 88:e0197921. doi: 10.1128/aem.01979-21, PMID: 35108089 PMC8939312

[ref49] Ghaddar B. Naoum-Sawaya J. (2017). High dimensional data classification and feature selection using support vector machines. Eur. J. Oper. Res. 265, 993–1004. doi: 10.1016/j.ejor.2017.08.040

[ref50] Griekspoor P. Colles F. M. Mccarthy N. D. Hansbro P. M. Ashhurst-Smith C. Olsen B.. (2013). Marked host specificity and lack of phylogeographic population structure of *Campylobacter jejuni* in wild birds. Mol. Ecol. 22, 1463–1472. doi: 10.1111/mec.12144, PMID: 23356487 PMC3596980

[ref51] Gu W. Cui Z. Stroika S. Carleton H. A. Conrad A. Katz L. S.. (2023). Predicting food sources of *Listeria monocytogenes* based on genomic profiling using random Forest model. Foodborne Pathog. Dis. 20, 579–586. doi: 10.1089/fpd.2023.0046, PMID: 37699246

[ref52] Guiney D. G. Fierer J. (2011). The role of the spv genes in Salmonella pathogenesis. Front. Microbiol. 2:129. doi: 10.3389/fmicb.2011.00129, PMID: 21716657 PMC3117207

[ref53] Haddad N. Johnson N. Kathariou S. Métris A. Phister T. Pielaat A.. (2018). Next generation microbiological risk assessment-potential of omics data for hazard characterisation. Int. J. Food Microbiol. 287, 28–39. doi: 10.1016/j.ijfoodmicro.2018.04.015, PMID: 29703417

[ref54] Harris S. (2018). SKA: Split Kmer analysis toolkit for bacterial genomic epidemiology. bioRxiv [Preprint]. doi: 10.1101/453142

[ref55] Hopfield J. J. (1982). Neural networks and physical systems with emergent collective computational abilities. Proc. Natl. Acad. Sci. USA 79, 2554–2558. doi: 10.1073/pnas.79.8.2554, PMID: 6953413 PMC346238

[ref56] Im H. Hwang S. H. Kim B. S. Choi S. H. (2021). Pathogenic potential assessment of the Shiga toxin-producing *Escherichia coli* by a source attribution-considered machine learning model. Proc. Natl. Acad. Sci. USA 118:e2018877118. doi: 10.1073/pnas.2018877118, PMID: 33986113 PMC8157976

[ref57] Jaudou S. Deneke C. Tran M. L. Schuh E. Goehler A. Vorimore F.. (2022). A step forward for Shiga toxin-producing *Escherichia coli* identification and characterization in raw milk using long-read metagenomics. Microb. Genom. 8:mgen000911. doi: 10.1099/mgen.0.000911, PMID: 36748417 PMC9836091

[ref58] Jin Y. Johannissen L. O. Hay S. (2021). Predicting new protein conformations from molecular dynamics simulation conformational landscapes and machine learning. Proteins 89, 915–921. doi: 10.1002/prot.26068, PMID: 33629765

[ref59] John P. Varga C. Cooke M. Majowicz S. E. (2022). Incidence, demographic, and seasonal risk factors of infections caused by five major enteric pathogens, Ontario, Canada, 2010-2017. Foodborne Pathog. Dis. 19, 248–258. doi: 10.1089/fpd.2021.0034, PMID: 35049363

[ref60] Joseph L. A. W. L. K. F. Chen J. Tagg K. A. Bennett C. Caidi H. Folster J. P.. (2020). Comparison of molecular subtyping and antimicrobial resistance detection methods used in a large multistate outbreak of extensively drug-resistant *Campylobacter jejuni* infections linked to pet store puppies. J. Clin. Microbiol. 58, e00771–e00820. doi: 10.1128/JCM.00771-20 32719029 PMC7512158

[ref61] Jumper J. Evans R. Pritzel A. Green T. Figurnov M. Ronneberger O.. (2021). Highly accurate protein structure prediction with AlphaFold. Nature 596, 583–589. doi: 10.1038/s41586-021-03819-2, PMID: 34265844 PMC8371605

[ref62] Kingsley R. A. Kay S. Connor T. Barquist L. Sait L. Holt K. E.. (2013). Genome and transcriptome adaptation accompanying emergence of the definitive type 2 host-restricted *Salmonella enterica* serovar typhimurium pathovar. MBio 4, e00565–e00613. doi: 10.1128/mBio.00565-13, PMID: 23982073 PMC3760250

[ref63] Ko K. K. K. Chng K. R. Nagarajan N. (2022). Metagenomics-enabled microbial surveillance. Nat. Microbiol. 7, 486–496. doi: 10.1038/s41564-022-01089-w, PMID: 35365786

[ref64] Leekitcharoenphon P. Johansson M. H. K. Munk P. Malorny B. Skarzynska M. Wadepohl K.. (2021). Genomic evolution of antimicrobial resistance in *Escherichia coli*. Sci. Rep. 11:15108. doi: 10.1038/s41598-021-93970-7 34301966 PMC8302606

[ref65] Lin S. Sun M. Fitzgerald E. Hwang S. A. (2016). Did summer weather factors affect gastrointestinal infection hospitalizations in New York state? Sci. Total Environ. 550, 38–44. doi: 10.1016/j.scitotenv.2015.12.153 26803682

[ref66] Lu B. Leong H. W. (2016). Computational methods for predicting genomic islands in microbial genomes. Comput. Struct. Biotechnol. J. 14, 200–206. doi: 10.1016/j.csbj.2016.05.001, PMID: 27293536 PMC4887561

[ref67] Luo J. Cao Y. Barzilay R. (2019). Neural decipherment via minimum-cost flow: From Ugaritic to linear B. arXiv [Preprint]. doi: 10.48550/arXiv.1906.06718

[ref68] Lupolova N. Lycett S. J. Gally D. L. (2019). A guide to machine learning for bacterial host attribution using genome sequence data. Microb. Genom. 5:e000317. doi: 10.1099/mgen.0.000317, PMID: 31778355 PMC6939162

[ref69] Ma L. Chen L. Chou K. C. Lu X. (2021). *Campylobacter jejuni* antimicrobial resistance profiles and mechanisms determined using a Raman spectroscopy-based Metabolomic approach. Appl. Environ. Microbiol. 87:e0038821. doi: 10.1128/AEM.00388-21, PMID: 33837016 PMC8174766

[ref70] Maiden M. C. Bygraves J. A. Feil E. Morelli G. Russell J. E. Urwin R.. (1998). Multilocus sequence typing: a portable approach to the identification of clones within populations of pathogenic microorganisms. Proc. Natl. Acad. Sci. USA 95, 3140–3145. doi: 10.1073/pnas.95.6.3140, PMID: 9501229 PMC19708

[ref71] Malberg Tetzschner A. M. Johnson J. R. Johnston B. D. Lund O. Scheutz F. (2020). In silico genotyping of *Escherichia coli* isolates for Extraintestinal virulence genes by use of whole-genome sequencing data. J. Clin. Microbiol. 58:e01269-20. doi: 10.1128/JCM.01269-20, PMID: 32669379 PMC7512150

[ref72] Mason L. Baxter J. Bartlett P. L. Frean M (1999). Boosting algorithms as gradient descent. Neural Information Processing Systems.

[ref73] Mehrab Z. Mobin J. Tahmid I. A. Pachter L. Rahman A. (2020). Reference-free association mapping from sequencing reads using k-mers. Bio Protoc. 10:e3815. doi: 10.21769/BioProtoc.3815, PMID: 33659468 PMC7842384

[ref74] Mellmann A. Harmsen D. Cummings C. A. Zentz E. B. Leopold S. R. Rico A.. (2011). Prospective genomic characterization of the German enterohemorrhagic *Escherichia coli* O104:H4 outbreak by rapid next generation sequencing technology. PLoS One 6:e22751. doi: 10.1371/journal.pone.0022751, PMID: 21799941 PMC3140518

[ref75] Merlotti A. Manfreda G. Munck N. Hald T. Litrup E. Nielsen E. M.. (2020). Network approach to source attribution of *Salmonella enterica* Serovar typhimurium and its monophasic variant. Front. Microbiol. 11:1205. doi: 10.3389/fmicb.2020.01205 34354676 PMC8335978

[ref76] Moradigaravand D. Palm M. Farewell A. Mustonen V. Warringer J. Parts L. (2018). Prediction of antibiotic resistance in *Escherichia coli* from large-scale pan-genome data. PLoS Comput. Biol. 14:e1006258. doi: 10.1371/journal.pcbi.1006258, PMID: 30550564 PMC6310291

[ref77] Mughini-Gras L. Kooh P. Fravalo P. Augustin J.-C. Guillier L. David J.. (2019). Critical orientation in the jungle of currently available methods and types of data for source attribution of foodborne diseases. Front. Microbiol. 10:2578. doi: 10.3389/fmicb.2019.02578, PMID: 31798549 PMC6861836

[ref78] Munck N. Njage P. M. K. Leekitcharoenphon P. Litrup E. Hald T. (2020). Application of whole-genome sequences and machine learning in source attribution of *Salmonella Typhimurium*. Risk Anal. 40, 1693–1705. doi: 10.1111/risa.13510, PMID: 32515055 PMC7540586

[ref79] Octavia S. Wang Q. Tanaka M. M. Kaur S. Sintchenko V. Lan R. (2015). Delineating community outbreaks of *Salmonella enterica* serovar typhimurium by use of whole-genome sequencing: insights into genomic variability within an outbreak. J. Clin. Microbiol. 53, 1063–1071. doi: 10.1128/JCM.03235-14, PMID: 25609719 PMC4365247

[ref80] Parsons B. N. Humphrey S. Salisbury A. M. Mikoleit J. Hinton J. C. Gordon M. A.. (2013). Invasive non-typhoidal *Salmonella typhimurium* ST313 are not host-restricted and have an invasive phenotype in experimentally infected chickens. PLoS Negl. Trop. Dis. 7:e2487. doi: 10.1371/journal.pntd.0002487, PMID: 24130915 PMC3794976

[ref81] Payne M. Octavia S. Luu L. D. W. Sotomayor-Castillo C. Wang Q. Tay A. C. Y.. (2021). Enhancing genomics-based outbreak detection of endemic *Salmonella enterica* serovar typhimurium using dynamic thresholds. Microb. Genom. 7:000310. doi: 10.1099/mgen.0.000310, PMID: 31682222 PMC8627665

[ref82] Pearce M. E. Alikhan N. F. Dallman T. J. Zhou Z. Grant K. Maiden M. C. J. (2018). Comparative analysis of core genome MLST and SNP typing within a European Salmonella serovar Enteritidis outbreak. Int. J. Food Microbiol. 274, 1–11. doi: 10.1016/j.ijfoodmicro.2018.02.023, PMID: 29574242 PMC5899760

[ref83] Pightling A. W. Pettengill J. B. Luo Y. Baugher J. D. Rand H. Strain E. (2018). Interpreting whole-genome sequence analyses of foodborne Bacteria for regulatory applications and outbreak investigations. Front. Microbiol. 9:1482. doi: 10.3389/fmicb.2018.01482 30042741 PMC6048267

[ref84] Pijnacker R. Dallman T. J. Tijsma A. S. L. Hawkins G. Larkin L. Kotila S. M.. (2019). An international outbreak of *Salmonella enterica* serotype Enteritidis linked to eggs from Poland: a microbiological and epidemiological study. Lancet Infect. Dis. 19, 778–786. doi: 10.1016/S1473-3099(19)30047-7, PMID: 31133519

[ref85] Power R. A. Parkhill J. De Oliveira T. (2017). Microbial genome-wide association studies: lessons from human GWAS. Nat. Rev. Genet. 18, 41–50. doi: 10.1038/nrg.2016.132, PMID: 27840430

[ref86] Quang D. Xie X. (2016). DanQ: a hybrid convolutional and recurrent deep neural network for quantifying the function of DNA sequences. Nucleic Acids Res. 44:e107. doi: 10.1093/nar/gkw226, PMID: 27084946 PMC4914104

[ref87] Rabsch W. Andrews H. L. Kingsley R. A. Prager R. Tschäpe H. Adams L. G.. (2002). *Salmonella enterica* serotype typhimurium and its host-adapted variants. Infect. Immun. 70, 2249–2255. doi: 10.1128/IAI.70.5.2249-2255.2002, PMID: 11953356 PMC127920

[ref88] Ren J. Ahlgren N. A. Lu Y. Y. Fuhrman J. A. Sun F. (2017). VirFinder: a novel k-mer based tool for identifying viral sequences from assembled metagenomic data. Microbiome 5:69. doi: 10.1186/s40168-017-0283-5 28683828 PMC5501583

[ref89] Saber M. M. Shapiro B. J. (2020). Benchmarking bacterial genome-wide association study methods using simulated genomes and phenotypes. Microb. Genom. 6:e000337. doi: 10.1099/mgen.0.000337, PMID: 32100713 PMC7200059

[ref90] Sanaa M. Pouillot R. Vega F. G. Strain E. Van Doren J. M. (2019). GenomeGraphR: a user-friendly open-source web application for foodborne pathogen whole genome sequencing data integration, analysis, and visualization. PLoS One 14:e0213039. doi: 10.1371/journal.pone.0213039, PMID: 30818354 PMC6394949

[ref91] Saund K. Snitkin E. S. (2020). Hogwash: three methods for genome-wide association studies in bacteria. Microb. Genom. 6:mgen000469. doi: 10.1099/mgen.0.000469, PMID: 33206035 PMC7725327

[ref92] Schjørring S. Gillesberg Lassen S. Jensen T. Moura A. Kjeldgaard J. S. Müller L.. (2017). Cross-border outbreak of listeriosis caused by cold-smoked salmon, revealed by integrated surveillance and whole genome sequencing (WGS), Denmark and France, 2015 to 2017. Eur. Secur. 22, 17–00762. doi: 10.2807/1560-7917.ES.2017.22.50.17-00762 PMC5743096 29258647

[ref93] Sephton-Clark P. Tenor J. L. Toffaletti D. L. Meyers N. Giamberardino C. Molloy S. F.. (2022). Genomic variation across a clinical Cryptococcus population linked to disease outcome. MBio 13:e0262622. doi: 10.1128/mbio.02626-22, PMID: 36354332 PMC9765290

[ref94] Sheppard S. K. Cheng L. Méric G. De Haan C. P. Llarena A. K. Marttinen P.. (2014). Cryptic ecology among host generalist *Campylobacter jejuni* in domestic animals. Mol. Ecol. 23, 2442–2451. doi: 10.1111/mec.12742, PMID: 24689900 PMC4237157

[ref95] Sheppard S. K. Jolley K. A. Maiden M. C. (2012). A gene-by-gene approach to bacterial population genomics: whole genome MLST of Campylobacter. Genes (Basel) 3, 261–277. doi: 10.3390/genes3020261, PMID: 24704917 PMC3902793

[ref96] Siguier P. Perochon J. Lestrade L. Mahillon J. Chandler M. (2006). ISfinder: the reference centre for bacterial insertion sequences. Nucleic Acids Res. 34, D32–D36. doi: 10.1093/nar/gkj014, PMID: 16381877 PMC1347377

[ref97] Smid J. H. Mughini Gras L. De Boer A. G. French N. P. Havelaar A. H. Wagenaar J. A.. (2013). Practicalities of using non-local or non-recent multilocus sequence typing data for source attribution in space and time of human campylobacteriosis. PLoS One 8:e55029. doi: 10.1371/journal.pone.0055029, PMID: 23405107 PMC3566096

[ref98] Svahn A. J. S. C. J. E. Chang S. L. Rockett R. J. Sim E. M. Cliff O. M. Wang Q.. (2023). Pangenome analysis of a *Salmonella Enteritidis* population links a major outbreak to a Gifsy-1-like prophage containing anti-inflammatory gene gogB. Microbiol. Spectr 11:e0279122. doi: 10.1128/spectrum.02791-22 36916949 PMC10100743

[ref99] Swaminathan B. Barrett T. J. Hunter S. B. Tauxe R. V. (2001). PulseNet: the molecular subtyping network for foodborne bacterial disease surveillance, United States. Emerg. Infect. Dis. 7, 382–389. doi: 10.3201/eid0703.017303, PMID: 11384513 PMC2631779

[ref100] Szarvas J. Bartels M. D. Westh H. Lund O. (2021). Rapid open-source SNP-based clustering offers an alternative to Core genome MLST for outbreak tracing in a hospital setting. Front. Microbiol. 12:636608. doi: 10.3389/fmicb.2021.636608, PMID: 33868194 PMC8047125

[ref101] Tanui C. K. Benefo E. O. Karanth S. Pradhan A. K. (2022). A machine learning model for food source attribution of *Listeria monocytogenes*. Pathogens 11:691. doi: 10.3390/pathogens11060691, PMID: 35745545 PMC9230378

[ref103] Tin Kam H. (1995). “Random decision forests” in *Proceedings of 3rd International Conference on Document Analysis and Recognition*. vol.1, 14–16 August, 1995. 278–282.

[ref104] Tiwari S. K. Van Der Putten B. C. L. Fuchs T. M. Vinh T. N. Bootsma M. Oldenkamp R.. (2023). Genome-wide association reveals host-specific genomic traits in *Escherichia coli*. BMC Biol. 21:76. doi: 10.1186/s12915-023-01562-w 37038177 PMC10088187

[ref105] Tyson G. W. Chapman J. Hugenholtz P. Allen E. E. Ram R. J. Richardson P. M.. (2004). Community structure and metabolism through reconstruction of microbial genomes from the environment. Nature 428, 37–43. doi: 10.1038/nature02340, PMID: 14961025

[ref106] Uelze L. Grützke J. Borowiak M. Hammerl J. A. Juraschek K. Deneke C.. (2020). Typing methods based on whole genome sequencing data. One Health Outlook 2:3. doi: 10.1186/s42522-020-0010-1 33829127 PMC7993478

[ref107] Uffelmann E. Huang Q. Q. Munung N. S. De Vries J. Okada Y. Martin A. R.. (2021). Genome-wide association studies. Nat. Rev. Methods Primers 1:59. doi: 10.1038/s43586-021-00056-9

[ref108] Van Belkum A. Melchers W. J. Ijsseldijk C. Nohlmans L. Verbrugh H. Meis J. F. (1997). Outbreak of amoxicillin-resistant *Haemophilus influenzae* type b: variable number of tandem repeats as novel molecular markers. J. Clin. Microbiol. 35, 1517–1520. doi: 10.1128/jcm.35.6.1517-1520.1997, PMID: 9163472 PMC229777

[ref109] Van Den Beld M. J. C. V. D. Z. K. Verbruggen A. Zutt I. Wolthuis R. Van Der Putten B. Bosch T. (2023). Twelfth external quality assessment scheme for Salmonella typing. ECDC.

[ref110] Van Kempen M. Kim S. S. Tumescheit C. Mirdita M. Lee J. Gilchrist C. L. M.. (2024). Fast and accurate protein structure search with Foldseek. Nat. Biotechnol. 42, 243–246. doi: 10.1038/s41587-023-01773-0, PMID: 37156916 PMC10869269

[ref111] Verschuuren T. Bosch T. Mascaro V. Willems R. Kluytmans J. (2022). External validation of WGS-based antimicrobial susceptibility prediction tools, KOVER-AMR and ResFinder 4.1, for *Escherichia coli* clinical isolates. Clin. Microbiol. Infect. 28, 1465–1470. doi: 10.1016/j.cmi.2022.05.024, PMID: 35662642

[ref112] Wainaina L. Merlotti A. Remondini D. Henri C. Hald T. Njage P. M. K. (2022). Source attribution of human Campylobacteriosis using whole-genome sequencing data and network analysis. Pathogens 11:645. doi: 10.3390/pathogens11060645, PMID: 35745499 PMC9229307

[ref113] Wheeler N. E. Gardner P. P. Barquist L. (2018). Machine learning identifies signatures of host adaptation in the bacterial pathogen *Salmonella enterica*. PLoS Genet. 14:e1007333. doi: 10.1371/journal.pgen.1007333, PMID: 29738521 PMC5940178

[ref114] Woodcock D. J. Krusche P. Strachan N. J. C. Forbes K. J. Cohan F. M. Méric G.. (2017). Genomic plasticity and rapid host switching can promote the evolution of generalism: a case study in the zoonotic pathogen Campylobacter. Sci. Rep. 7:9650. doi: 10.1038/s41598-017-09483-9 28851932 PMC5575054

[ref115] Yang Z. Zeng X. Zhao Y. Chen R. (2023). AlphaFold2 and its applications in the fields of biology and medicine. Signal Transduct. Target. Ther. 8:115. doi: 10.1038/s41392-023-01381-z 36918529 PMC10011802

[ref116] Yoon S. H. Park Y.-K. Kim J. F. (2014). PAIDB v2.0: exploration and analysis of pathogenicity and resistance islands. Nucleic Acids Res. 43, D624–D630. doi: 10.1093/nar/gku985 25336619 PMC4384037

[ref117] Zankari E. Allesøe R. Joensen K. G. Cavaco L. M. Lund O. Aarestrup F. M. (2017). PointFinder: a novel web tool for WGS-based detection of antimicrobial resistance associated with chromosomal point mutations in bacterial pathogens. J. Antimicrob. Chemother. 72, 2764–2768. doi: 10.1093/jac/dkx217, PMID: 29091202 PMC5890747

[ref118] Zhang S. Li S. Gu W. Den Bakker H. Boxrud D. Taylor A.. (2019). Zoonotic source attribution of *Salmonella enterica* serotype typhimurium using genomic surveillance data, United States. Emerg. Infect. Dis. 25, 82–91. doi: 10.3201/eid2501.180835, PMID: 30561314 PMC6302586

[ref119] Zvyagin M. Brace A. Hippe K. Deng Y. Zhang B. Bohorquez C. O.. (2022). GenSLMs: genome-scale language models reveal SARS-CoV-2 evolutionary dynamics. bioRxiv [Preprint]. doi: 10.1101/2022.10.10.511571

